# Enhancing global disaster preparedness: A scoping review of the current integration of situational awareness and disaster mindset in healthcare education

**DOI:** 10.3934/publichealth.2025038

**Published:** 2025-08-05

**Authors:** Amir Khorram-Manesh, Gulcan Taskiran Eskici, Lesley Gray

**Affiliations:** 1 Department of Surgery, Institute of Clinical Sciences, Sahlgrenska Academy, University of Gothenburg, 413 45, Gothenburg, Sweden; 2 Center for Disaster Medicine, University of Gothenburg, 405 30, Gothenburg, Sweden; 3 Gothenburg Emergency Medicine Research Group (GEMREG), Sahlgrenska University Hospital, 413 45, Gothenburg, Sweden; 4 Department of Nursing Administration, Faculty of Health Sciences, Ondokuz Mayis University, 57270, Samsun, Türkiye; 5 Department of Primary Health Care & General Practice, Faculty of Medicine, University of Otago, 6242, Wellington, New Zealand

**Keywords:** disaster, disaster medicine, education, mindset, program, situational awareness, healthcare professionals

## Abstract

Current disaster medicine programs and simulation exercises often fail to adequately incorporate crucial elements such as situational awareness and a disaster mindset. This gap in training can significantly impact the effectiveness of healthcare professionals' responses during real-world disasters and public health emergencies. In this review, we examined the literature to explore the critical role of situational awareness and a disaster mindset in enhancing healthcare provider preparedness for disaster events, proposing the integration of innovative technologies, such as virtual and augmented reality, to create immersive simulations that foster situational awareness and a resilient disaster mindset. Addressing this educational gap will improve healthcare professionals' confidence and optimize patient outcomes during crises.

## 1. Introduction

Over the past five decades, disaster medicine education has evolved from a basic focus on immediate response to a comprehensive, multidisciplinary field, partly due to the increased severity and globalization of incidents. In the 1970s and 1980s, disaster medicine education concentrated on basic emergency response and triage, with limited formal training programs. The 1972 Munich Olympics attack exposed the vulnerabilities of large-scale events, leading to a significant shift in security planning. It highlighted the need for comprehensive measures like intelligence gathering, risk assessment, and coordinated response plans. The attack also revealed gaps in emergency response, particularly in handling hostage situations and mass casualties, prompting improvements in interagency communication and cooperation. This event underscored the need for specialized training in managing terrorist attacks, including blast injuries, chemical and biological threats, and psychological trauma [1],[2].

The 1985 Mexico City earthquake highlighted the need for disaster preparedness, including early warning systems, evacuation plans, and seismic-resistant building codes. It revealed gaps in medical response and education infrastructure, leading to advancements in mass casualty management and the importance of well-trained medical personnel. The international response demonstrated the value of global collaboration in disaster relief, emphasizing better coordination and disaster-resistant healthcare facilities [2]–[4].

The 1990s saw the formalization of disaster medicine as a distinct field. Universities and medical schools introduced specialized courses and degrees, and diverse disaster medicine organizations were established to standardize training and certification [2]. During the 2000s, disaster medicine education integrated more closely with emergency management, adopting a multidisciplinary approach that included public health, logistics, and communication. Technological advancements, such as simulation-based training and online learning platforms, revolutionized the field [5].

The 2010s brought a global perspective to disaster medicine education, driven by frequent global disasters like the 2010 Haiti earthquake and the 2011 Fukushima nuclear disaster. Both disasters had significant global implications, shaping humanitarian responses, economic policies, and discussions on safety and preparedness, and serve as reminders of the importance of international cooperation and proactive measures in mitigating the impacts of natural hazards and human-induced disasters [6],[7]. Disaster medicine provides the specialized medical knowledge and skills, while humanitarian response provides the broader framework and operational context within which these medical interventions are delivered to populations in crisis [8]. Since then, training programs have incorporated international standards and best practices, expanding the curriculum to cover disaster preparedness, risk reduction, and recovery, including mental health and community resilience [9].

Finally, in the 2020s, the growing impact of climate change led to the inclusion of training on responding to climate-related disasters [10]. The evolution of disaster medicine education to include public health and climate health reflects a growing recognition of the interconnectedness of these fields, including the spread of infectious diseases, environmental hazards, and the disruption of essential health services. Disaster medicine education increasingly incorporates public health principles such as epidemiology, population health, and health promotion. This enables healthcare professionals to address the broader health needs of affected populations, not just individual patients [11].

Eleven core competencies for disaster medicine and public health were identified by Walsh et al. [12] for the American Medical Association in 2012. Besides those competencies, situational awareness (SA) and a disaster mindset (DMS) are critical competencies requiring adaptive application contingent on the evolving scale and nature of emergency incidents [13]–[16]. SA refers to understanding the immediate environment, evolving events, and potential future impacts to effectively inform decision-making and action [17], while a DSA embodies a proactive and adaptive cognitive state that anticipates potential catastrophic events, prioritizes decisive action under pressure, and maintains a focus on effective response and recovery efforts [18]. Disasters and emergencies are dynamic events capable of escalating along a continuum from localized multiple-casualty incidents (MUCIs) to widespread mass-casualty incidents (MCIs), and to catastrophic disaster casualty (DC) events, often driven by resource limitations and infrastructural insufficiencies or damage.

A MUCI is characterized by several patients exceeding routine local resources yet remains geographically contained and manageable within the existing emergency response framework [19],[20]. In such scenarios, SA is acutely focused on the immediate environment, and the specific needs of a limited number of casualties, necessitating rapid information processing for swift decision-making regarding patient stabilization and transport. The corresponding DMS emphasizes prompt, efficient operational actions and optimized resource allocation within a defined operational area [18]. Effective MUCI management is vital to evidence-based healthcare strategies in emergency preparedness [21].

An MCI can develop from the escalation of a MUCI due to factors such as a sudden surge in casualties, complex injuries surpassing local capabilities, infrastructural damage impeding access, concurrent emergencies straining resources, or a rapid expansion of the affected geographical zone, collectively leading to resource overwhelm [22],[23]. SA in MCIs demands a broader comprehension of the operational environment, encompassing casualty location and numbers alongside resource availability, requiring coordinated efforts among multiple agencies and the assimilation of accurate and prompt information for strategic command decisions. The DMS in MCIs prioritizes strategic planning, establishing clear command structures and standardized triage protocols, and prioritizing actions that aim to maximize benefit for the greatest number of patients, often involving complex and ethically challenging resource allocation choices [23],[24].

In contrast, SA in disasters and complex emergencies broadens significantly to encompass the affected geographic area, the magnitude of infrastructural damage, and long-term societal and environmental ramifications, necessitating comprehensive data collection and sophisticated analysis for a holistic crisis understanding. Effective SA in disasters mandates robust collaboration among diverse entities, including healthcare providers, emergency services, governmental bodies, and community organizations, with seamless information and resource sharing being paramount [10],[24].

The DMS in large-scale disasters emphasizes resilience and adaptability, requiring responders to be prepared for protracted operations and the management of continuously evolving challenges. Crucially, engaging the affected community and cultivating strong interpersonal relationships are essential for effective disaster response and recovery, encompassing public education, trust-building, and widespread preparedness [25],[26]. Table 1 shows the transition between multiple-, mass-, and disaster casualties and the necessary aspects and characteristics. This transformation reflects the increasing complexity and frequency of disasters and emergencies, necessitating a coordinated and informed approach to saving lives and mitigating impacts. Consequently, there must be a strong emphasis on interdisciplinary and transdisciplinary collaboration involving healthcare professionals, emergency responders, policymakers, and community leaders in the education and practice of disaster science. Continuous research and feedback from real-world disaster responses ensure that disaster medicine education remains relevant and effective [27],[28].

Despite advancements in the field, the current state of disaster medicine education is characterized by a notable lack of global homogeneity in curriculum content and available opportunities [29]–[31]. This heterogeneity stems from the inherent diversity in hazard profiles, etiological factors, resource availability, experiential learning, and specialized expertise across different regions. Prior investigations have consistently underscored the imperative for a standardized pedagogical framework in disaster medicine education while highlighting significant limitations within existing training methodologies [29]–[31].

Although progress has been made in multi-agency training initiatives, educational programs, and competency-based frameworks designed to enhance intercultural, interdisciplinary, and transdisciplinary integration [2],[8],[27]–[30], the inherent cultural and educational heterogeneities often result in a suboptimal focus on individual professional development, potentially fostering resistance to novel approaches [32]. Furthermore, deficiencies in incorporating SA and DSM cultivation within training and education [33] may predispose individuals and organizations to conflictual dynamics during disaster response operations [32],[34]. This phenomenon may elucidate the inherent challenges in transitioning operational paradigms from the more frequently encountered multi-casualty incident scenario to the complexities of a large-scale disaster event [35].

The transition of disaster medicine, primarily focusing on medical and health-related aspects of disasters, to disaster management, focusing on overall coordinated efforts to mitigate, prepare for, respond to, and recover from disasters, makes disaster medicine a crucial component of disaster management. Effective disaster management relies on a well-planned and executed disaster medicine response, but it also encompasses many other non-medical aspects necessary for a comprehensive and successful approach to disasters [36]. Thus, we explore the importance of SA and DMS in disaster and emergency management, investigate whether the current educational initiatives deliver these two concepts to ensure that healthcare professionals are better prepared for the transition and complexities of real-world disaster scenarios.

**Table 1.** Characteristics of and differences between multiple casualties, mass casualties, and disaster casualties [19]–[24].

**Aspect**	**Multiple casualty**	**Mass casualty**	**Disaster casualty**
Definition	Incidents with a moderate number of casualties, and manageable with local resources.	Incidents with a large number of casualties overwhelm local emergency response resources.	Incidents resulting in casualties due to natural or human-made disasters.
Scale	There are a moderate number of casualties.	There were a large number of casualties.	This varies widely, from small to large scale.
Resource Impact	May be managed with local resources.	Overwhelms local resources, requiring external support.	The impact on resources varies depending on the disaster.
Nature of Incident	Typically, controlled environments like traffic accidents or smaller-scale fires.	High-impact events like terrorist attacks, mass shootings, or large-scale accidents.	Wide range of events, including natural and human-made disasters.
Response Focus	Efficient triage, treatment, and transport within a controlled environment.	Rapid triage, treatment, and stabilization of a large number of patients.	Comprehensive response including search and rescue, medical care, and long-term recovery.
Triage Systems	Standard systems like START.	Advanced systems like SALT and MUCC.	A combination of triage systems may be used depending on the scale and nature of the disaster.
Coordination	Local coordination among emergency services, hospitals, and other stakeholders.	Extensive coordination among multiple agencies, including federal and state emergency management.	Broad coordination involving various agencies and long-term recovery efforts.
Environmental Factors	More controlled environments.	Complex, chaotic environments with additional hazards.	May involve hazardous environments complicating response efforts.

Note: START: Simple Triage and Rapid Treatment; SALT: Sort, Assess, Life-Saving Interventions, Treatment/Transport; MUCC: Model Uniform Core Criteria for Mass Casualty Triage.

## 2. Materials and methods

In this review, we aim to map the literature on integrating SA and DMS in disaster medicine education programs globally. Our objective is to identify the available evidence types, clarify key concepts, examine how SA and DMS are being addressed (or not) in educational contexts, and highlight potential research gaps. This broad mapping exercise will encompass diverse literature, including experiential reports, educational frameworks, and simulation methodologies [37]. The PRISMA-ScR framework was applied to this study (see supplementary file).

### 2.1. Research question

What is the extent and nature of integrating SA and DMS into current disaster medicine education programs?

### 2.2. Framework

The scope of this review was defined using the PCC framework: a) Population: Healthcare professionals (students, trainees, practicing) involved in disaster medicine education, including medical, nursing, allied health, and public health; b) Concept: Integration or consideration of SA and/or DMS; and c) Context: Disaster medicine programs, curricula, simulation exercises, educational frameworks, and related pedagogical approaches.

### 2.3. Search strategy

A comprehensive and iterative search strategy was developed to identify relevant publications in electronic databases and grey literature sources. The following databases were systematically searched: PubMed, Scopus, and Web of Science (WoS). No limitations were included for year of publication to date (2025). For the search strategy, we utilized a combination of keywords and Medical Subject Headings (MeSH terms) where applicable, aligned with the PCC framework. The core search terms included:

- Situational Awareness (SA): “Situational Awareness,” “Environmental Awareness,” “Crisis Awareness,” “Threat Recognition,” AND “Sensemaking.”
- Disaster Mindset (DMS): “Disaster Mindset,” “Disaster Preparedness,” “Emergency Preparedness Education,” “Psychological Preparedness,” “Resilience,” AND “Preparedness.”
- Disaster Medicine Programs/Education: “Disaster Medicine” AND (“Education” OR “Program” OR “Training” OR “Curriculum” OR “Simulation”).

### 2.4. Search strings

Two primary search strings were used for each database to isolate the integration of SA and DMS within disaster medicine programs, as shown in Table 2.

Google Scholar and the Google search engine were also utilized with similar keyword combinations to further broaden the search and capture grey literature. The search results from the first 100 relevant hits were screened.

**Table 2.** Search strings used in each database, PubMed, Scopus, and WoS.

**Database**	**Search Strings**
PubMed	“Disaster Medicine” AND (“Education” OR “Program” OR “Training” OR “Curriculum” OR “Simulation”) AND “Situational Awareness”
PubMed	“Disaster Medicine” AND (“Education” OR “Program” OR “Training” OR “Curriculum” OR “Simulation”) AND (“Disaster Mindset” OR “Disaster Preparedness Education” OR “Emergency Preparedness Education” OR “Psychological Preparedness”)
Scopus	“Disaster Medicine” AND (“Education” OR “Program” OR “Training” OR “Simulation”) AND “Situational Awareness”
Scopus	“Disaster Medicine” AND (“Education” OR “Program” OR “Training” OR “Curriculum” OR “Simulation”) AND (“Disaster Mindset” OR “Preparedness Education” OR “Psychological Preparedness”)
Web of Science	(“Disaster Medicine” AND (“Education” OR “Program” OR “Training” OR “Curriculum” OR “Simulation”) AND “Situational Awareness”)
Web of Science	(“Disaster Medicine” AND (“Education” OR “Program” OR “Training” OR “Curriculum” OR “Simulation”) AND (“Disaster Mindset” OR “Preparedness Education” OR “Psychological Preparedness”)

### 2.5. Inclusion and exclusion criteria

#### 2.5.1. Studies were included if they met the following criteria

- *Language:* Published in English.
- *Focus:* Explicitly discussed or addressed the integration or consideration of situational awareness AND/OR disaster mindset within the context of disaster medicine education, programs, or simulation exercises for healthcare professionals (including medical, nursing, allied health, and public health).
- *Context:* Relevant to disasters and public health emergencies.
- *Publication Type:* All available publications meeting the inclusion criteria were included, including review articles, conceptual papers, educational reports, guidelines, and case studies describing SA, DMS, and their incorporation into educational interventions.

#### 2.5.2. Studies were excluded if they

- Focused solely on clinical aspects of disaster response without addressing educational methodologies for SA or DMS.
- Considered the use of technology in disaster response without a clear link to the educational aspects of SA or DMS.
- Related to disaster preparedness for the general public or non-healthcare professionals.
- Did not meet the inclusion criteria or were published in languages other than English.
- They were purely theoretical without any clear connection to practical educational applications in disaster medicine.

### 2.6. Study selection process

One of the authors performed the search under the guidance of a librarian. The identified records from the database searches were imported into a Word document. Duplicates were removed through manual review. The study selection process involved two stages:

#### 2.6.1. Stage 1

Title and Abstract Screening: All authors independently screened the titles and abstracts of all unique records, based on the pre-defined inclusion and exclusion criteria. Uncertainties or potentially relevant articles proceeded to the full-text review stage.

#### 2.6.2. Stage 2

Full-Text Review: Full-text versions of the selected articles were retrieved and assessed against the inclusion and exclusion criteria by all authors. Reasons for exclusion at this stage were documented. A record of the screening and selection process was maintained.

### 2.7. Data extraction

A structured data extraction form was developed to extract relevant information from the included full-text articles. The following data points were targeted:

- Study characteristics (author, year, publication type, and country/region).
- Description of the disaster medicine education program or simulation exercise.
- How situational awareness was defined or described (if at all).
- Specific methods or strategies used to incorporate or address situational awareness.
- How the disaster mindset was defined or described (if at all).
- Specific methods or strategies used to cultivate or address the disaster mindset (e.g., psychological preparedness training, leadership exercises, ethical considerations).
- Reported outcomes or assessments related to SA or DMS (if any).
- Identified barriers or facilitators to incorporating SA or DMS.
- Key themes or conclusions related to the integration of SA and DMS.

### 2.8. Data synthesis and content analysis

The extracted data were synthesized using a narrative approach. This involved identifying and describing common themes, patterns, and variations in how SA and DMS are currently integrated (or not integrated) into disaster medicine education. Gaps in the literature and potential areas for improvement were highlighted. The synthesis was structured around the key aspects of the research question, providing a comprehensive overview of the current landscape and informing recommendations for enhancing disaster medicine training [38]. The thematic presentation focused on SA and DMS. A deductive content analysis was performed using two already selected thematic topics, SA and DMS, sorting the information into these predefined categories to see how well the data fits into those categories and test our hypothesis [39].

## 3. Results

**Figure 1.**
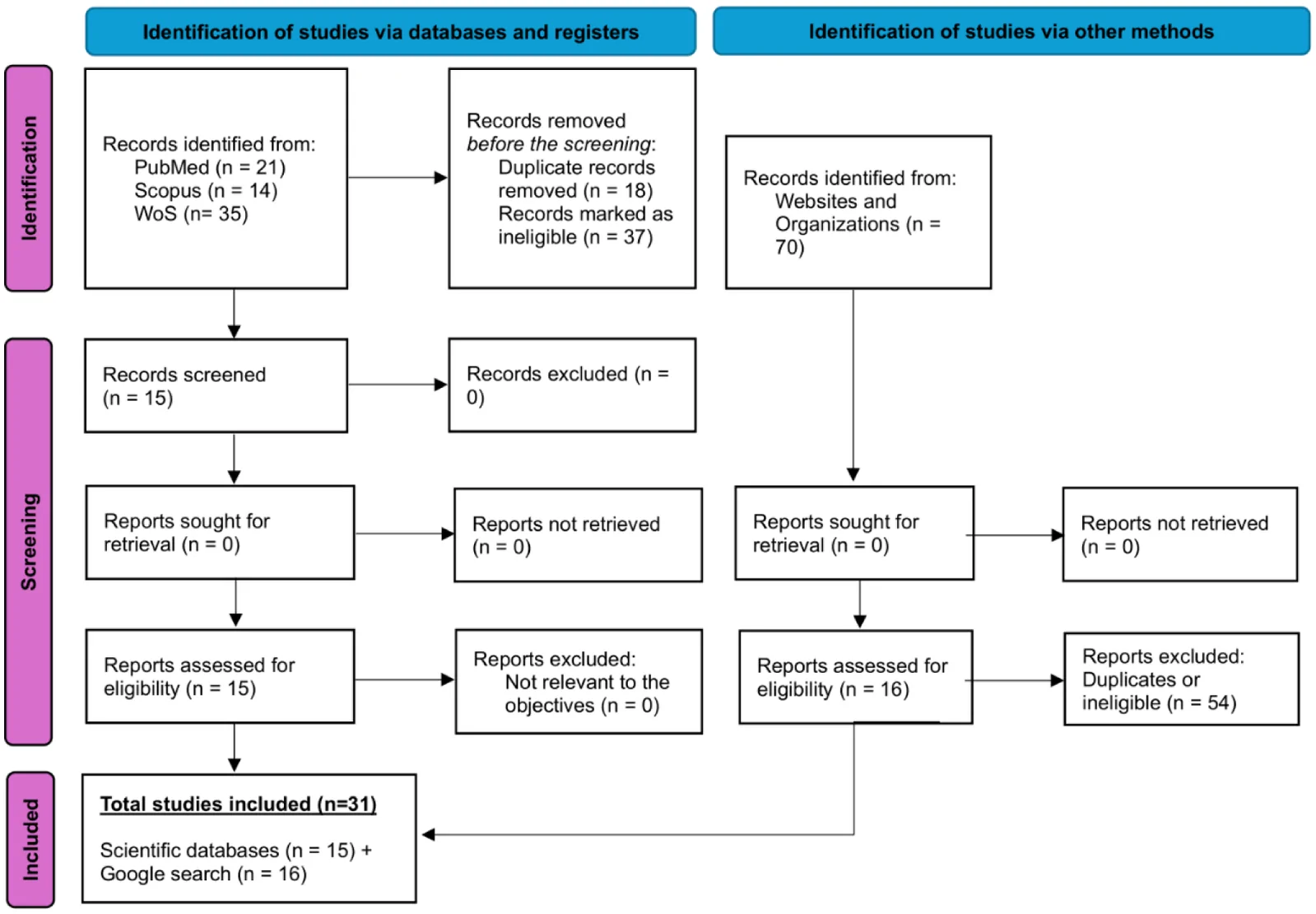
PRISMA flowchart for scoping reviews in the literature selection process.

The search resulted in 70 studies: PubMed (*n* = 21), Scopus (*n* = 14), and WoS (*n* = 35), of which 18 were duplicates, 37 did not meet the inclusion criteria, and 15 were included in the final stage for a review of the scientific data. Figure 1 shows the PRISMA literature selection process of included studies.

The first 70 hits from Google Scholar and Google engine searches for grey literature were thoroughly reviewed, and 16 studies discussing SA and DMS were included using the same criteria. The sum of articles included in this study was 31 [40]–[70]. The outcome was categorized into two subgroups: SA and DMS.

The included studies emphasize the foundational role of SA and DMS for effective interdisciplinary collaboration in emergency response, enabling proactive and adaptable decision-making, which are crucial for strategic, adapted, and resilient outcomes. Rather than being innate, the cultivation of SA and DSM occurs through dedicated training, integration of advanced technology, and the establishment of a collaborative culture [40]–[43]. Table 3 highlights other potential definitions and synonyms that might have been used for SA and DMS in the literature. The scientific studies included are summarized in Table 4.

**Table 3.** Key definitions and characteristics of situational awareness and a disaster mindset.

**Concept**	**Key components/Characteristics**
**Situational Awareness**	Perception, Comprehension, Projection, Understanding, Analyzing, Anticipating, Responding
**Disaster mindset**	Proactive, Preemptive, Resilient, Prepared, Psychological Prowess, Foresight, Coping

### 3.1. The critical role of SA

SA is vital for prompt and informed decision-making, effective communication, and efficient resource allocation [17],[18],[40]–[42]. A deficit in SA can precipitate adverse consequences, leading to poor decision-making, jeopardizing safety, increasing mistakes, reducing performance and efficiency, communication breakdown, and loss of trust and confidence. Ultimately, poor SA undermines safe and effective interaction with the environment, causing negative outcomes across various fields [43]. This dynamic process of SA necessitates a critical and continuous assessment of potential impacting factors and anticipation of future adversaries [44],[45]. Technological advancements are pivotal in augmenting SA capabilities; for instance, Unmanned Aerial Vehicles (UAVs) can provide comprehensive aerial perspectives, thereby enhancing situational understanding [46]. Furthermore, the adaptability of strategies and insights from other high-stakes fields, such as military surgery, underscores the acquirable nature of SA and its potential for cross-domain application [47]. Research consistently demonstrates that targeted training and simulation exercises are effective in improving both SA and confidence among emergency responders [14], with consistent, practical training emerging as paramount for effective disaster response [15]. To ensure optimal outcomes, proactive engagement and education are essential for maintaining critical SA throughout a crisis [48].

Complementary to SA, DMS emphasizes preparedness, strategic planning, and effective decision-making under pressure [49]. This encompasses key attributes such as adaptability, resilience, collaboration, and continuous learning, collectively ensuring decisive action and coordinated responses. Conversely, a lack of well-developed DMS can significantly hinder effective disaster and emergency management [18],[50]. Moreover, consistent training and drills serve as effective mechanisms for cultivating a strong DMS, equipping professionals with the necessary competencies to manage resource scarcity, make rapid decisions under duress, and address the psychological impact of mass casualty events [15]. Given the inherently unpredictable nature of disasters, fostering a proactive disaster mindset, where professionals anticipate and prepare for potential scenarios, is crucial [51]. This mindset, coupled with adaptability, resilience, and shared SA, is essential for effective coordinated decision-making in dynamic crises [52]. Ultimately, training plays a transformative role in shaping mindsets, enabling effective action grounded in comprehensive SA and strategic decision-making processes [53]. Therefore, a holistic approach that strategically integrates technological advancements with targeted training initiatives is necessary to foster robust SA and proactive DMS at all levels within emergency response organizations, thereby significantly enhancing overall disaster preparedness and response capabilities.

**Table 4.** Synthesis of 15 scientific research papers on situational awareness and a disaster mindset.

**No., Paper title/Authors/ Year/Journal/ Ref number**	**Focus of research**	**Key findings related to SA**	**Key findings related to DMS**	**Methodology used**
1. A paramedic field supervisor's situational awareness in prehospital emergency care. Prehospital and disaster medicine, Norri-Sederholm et al, 2014, Disaster Med Public Health Prep [44]	SA of paramedic field supervisors in prehospital settings	SA is dynamic and relies on communication and experience. Challenges include information overload and time pressure.	Foresight, stress management, and problem-solving skills were observed.	Qualitative study using semi-structured interviews. Data was analyzed via qualitative content analysis to identify themes in their SA processes.
2. Comparison of Unmanned Aerial Vehicle Technology versus Standard Practice of Scene Assessment by Paramedic Students of a Mass-Gathering Event. Jain et al, 2021, Prehosp Disaster Med [46]	UAV technology for scene assessment at mass gatherings	UAV technology provided accurate, safe, and feasible scene assessment, though no statistical difference was observed in most variables compared to standard practice.	N/A	Randomized comparison study
3. Surgeon preparedness for mass casualty events: Adapting essential military surgical lessons for the home front. Remick et al, 2016. Am J Disaster Med [47]	Surgeon preparedness for mass casualty events	Highlights the importance of situational awareness in military Damage Control Surgery (mDCS) that can be adapted to civilian settings.	Integrating military surgical lessons into civilian medical education can enhance preparedness and foster interdisciplinary collaboration.	Description of a curricular teaching module and its outcomes
4. An Island-Wide Disaster Drill to Train the Next Generation of Anesthesiologists: The SIAARTI Academy Experience. Carenzo et al, 2021. Disaster Med Public Health Prep [51]	Disaster drill for anesthesiology residents	Drills provided a practical understanding of mass-casualty response principles; debriefing highlighted the need to switch from a clinical to a managerial role.	Participants appreciated the need to shift to a managerial role, indicating a development in their mindset towards disaster response.	Simulation exercise with debriefing
5. Towards a model for team learning in multidisciplinary crisis management teams. Van Der Haar et al, 2008, Int J Emerg Manag [54]	Team learning in crisis management teams	Effective performance requires connectivity and shared visions through communication, leading to shared SA and mental models; TMS is crucial.	Implicitly suggests that team learning contributes to a more prepared and effective team.	Literature review and conceptual model development
6. Increased Situation Awareness in Major Incidents—Radio Frequency Identification (RFID) Technique: A Promising Tool. Jokela et al, 2012, Prehosp Disaster Med [56]	Technology for situational awareness in mass-casualty incidents	RFID systems provide information on casualties significantly faster than traditional methods, improving the overall view of the situation and enhancing emergency readiness.	N/A	A simulation study comparing RFID with traditional methods
7. Mobile phones and short message service texts to collect situational awareness data during simulated public health critical events. Magee et al, 2011, Am J Disaster Med [57]	Using mobile phones for situational awareness in public health emergencies	SMS texting proved to be a quick and reliable method for collecting situational awareness data, and participants found it easy to use and effective for communication.	N/A	Pilot study using simulated public health events and SMS data collection
8. Rural hospital incident command leaders' perceptions of disaster preparedness. Murphy et al, 2025, BMC Emerg Med [58]	Disaster preparedness in rural hospitals	Situational awareness and decision-making were identified as key challenges for HICG leaders, particularly concerning fewer familiar threats.	HICG leaders generally felt confident but acknowledged gaps; preparedness was facilitated by training and mental readiness.	Qualitative study using interviews and focus groups
9. Common Challenges in Prehospital Management of Mass-Casualty Incidents: A Systematic Integrative Review. Hugelius and Becker, 2024, Prehosp Disaster Med [59]	Prehospital management of mass-casualty incidents	Developing and communicating situational awareness is a significant challenge; resilient response requires understanding and foreseeing medical consequences.	Emphasizes the need for mental preparation of EMS personnel and medical incident commanders to adapt beyond routine practices.	Systematic integrative review of case studies and reports
10. An active shooter in your hospital: a novel method to develop a response policy using in situ simulation and video framework analysis. Argintaru et al, 2021, Disaster Med Public Health Prep [60]	To develop a hospital active shooter response policy using in situ simulation and video analysis.	Simulations revealed SA challenges in initial information gathering, communication, environmental awareness, and adapting to the dynamic threat. Video analysis pinpointed SA failures impacting response.	Simulations highlighted varying stress responses, problem-solving approaches, levels of proactive behavior, and the role of debriefing in building resilience. Policy development aimed to instill a more prepared mindset.	A novel method combining in situ simulations of active shooter scenarios in a hospital, systematic video framework analysis of staff responses, iterative policy development based on findings, and qualitative data from debriefing sessions.
11. Building health care system capacity: training health care professionals in disaster preparedness health care coalitions. Walsh et al, 2015, Prehosp Disaster Med [61]	Training in Health Care Coalitions for disaster preparedness	Promising practices in HCCs include improving situational awareness, promoting planning, and enabling resource sharing.	Identifies training needs and challenges in HCCs, highlighting the importance of stakeholder engagement and prioritizing training.	Qualitative study using semi-structured interviews with HCC leaders
12. Non-technical skills needed by medical disaster responders—a scoping review. Westman et al, 2024, Scan J Trauma Resus Emerg Med [62]	Non-technical skills for disaster responders	Situational awareness is identified as one of the four most frequently mentioned non-technical skills essential for effective disaster response.	Highlights the lack of uniform terminology for skills and competence, suggesting a need for further research on defining and training non-technical skills.	A scoping review of the literature
13. Earthquake Preparedness and Knowledge of Recommended Self-Protective Actions: A Survey of Nursing Students. Longo, 2022, Disaster Med Public Health Prep [63]	Earthquake preparedness of nursing students	Assesses existing knowledge of self-protective actions.	Reveals a significant lack of personal disaster preparedness and misconceptions about self-protective actions, indicating a need to foster a preparedness mindset.	Descriptive cross-sectional survey
14. All hazards training: incorporating a catastrophe preparedness mindset into the dental school curriculum and professional practice. Glotzer et al, 2007, Dent Clin North Am [64]	Disaster preparedness in dental education	N/A	Advocates for integrating a catastrophe preparedness mindset into the dental school curriculum through integrated elements and a capstone course; emphasize interprofessional collaboration.	Conceptual paper based on experience at NYU College of Dentistry
15. Emergency Management and Preparedness Training for Youth (EMPTY): The Results of the First Swedish Pilot Study. Khorram-Manesh et al, 2018, Disaster Med Public Health Prep [66]	Emergency preparedness training for youth	Simulation training significantly increased students' personal and situational awareness.	The EMPTY program effectively raised youth awareness and preparedness, emphasizing mental readiness, collaboration, and understanding of consequences.	Simulation-based pilot study with pre-, and post-tests and observer evaluations

Note: UAV: unmanned aerial vehicle; TMS: Transactive memory system; HICG: Hospital Incident Command Group; HCC's: healthcare coalitions

In this context, the importance of team dynamics is highlighted by the 2008 study by van der Haar et al., which underscores the significance of team learning in bolstering the performance of crisis management teams [54]. These teams, often composed of individuals with diverse expertise, must rapidly coalesce into cohesive units, and their effectiveness relies on connectivity, facilitated by open communication leading to shared visions and intentions. This connectivity is supported by specific team-learning behaviors, face-to-face interaction, a Transactive Memory System (TMS), shared SA, and shared mental models of the task and team capabilities. Shared SA enables coordinated action, while shared mental models streamline collaboration and problem-solving [55]. A functional TMS ensures rapid access to necessary knowledge and skills, with the study proposing a model emphasizing connectivity, shared understanding, and TMS development as critical elements for improving crisis response effectiveness.

Norri-Sederholm and co-authors highlight the critical importance of strong SA for paramedic field supervisors in prehospital emergency care in their 2014 study [44]. These supervisors rely heavily on well-developed SA to coordinate EMS units and make decisions in complex situations. Their SA includes understanding the evolving event, environmental factors, capabilities, action patterns, anticipated decisions, their multiple roles, and how they build SA through multitasking and data integration. The study emphasizes formal training and experience for developing paramedic field supervisors' SA and offers insights for improving their work, training, and support systems.

Jokela et al.'s 2012 study explored using RFID and mobile phone technology to improve SA in mass-casualty incidents (MCIs). Recognizing communication and information management as key challenges where paper-based methods are slow and inaccurate, they tested an RFID prototype in simulated MCIs. The results showed the RFID system was faster and more accurate in managing casualty information, offering a more stable and user-friendly solution that enhanced overall understanding of MCI situations and medical emergency readiness [56].

Jain et al.'s 2022 research explored unmanned aerial vehicle (UAV) technology's potential to improve SA for medical incident commanders at mass-gathering events. Comparing UAVs with standard scene assessment by paramedic students, the study found UAVs to be a safe and feasible tool offering a beneficial aerial perspective that enhanced overall SA. While comparable to traditional methods in identifying ground-level details, the UAV's unique aerial view provided distinct advantages for comprehensive scene understanding, suggesting a valuable role in emergency medical response with further optimization needed [46].

The potential to leverage widely accessible technology for SA gathering is explored in Magee et al.'s 2011 study, which demonstrated the feasibility of using two-way SMS communication via mobile phones to gather timely and valuable SA data during simulated public health emergencies from a distributed population. The findings supported the utility of this approach for rapid information collection, recommending further research to optimize its use in broader public health emergency response scenarios [57].

However, the challenges and requirements for effective disaster and emergency management can vary significantly depending on the context, with resource scarcity and geographical isolation being significant factors, as highlighted by Murphy et al.'s 2025, who examined the perceptions of Hospital Incident Command Group leaders in rural northern Sweden regarding their hospitals' readiness for major incidents [58]. They stressed the need for enhanced SA, effective decision-making, and thorough risk assessments for good disaster and emergency management in these settings, recommending targeted training, better coordination, and stronger resource planning to improve rural healthcare disaster readiness.

Hugelius and Becker's 2024 highlighted key challenges in the prehospital phase of MCIs, including ensuring safety, developing and communicating SA (among responders and to facilities), managing communication and information, creating tailored plans, providing care in severe conditions, and establishing extended response strategies. They concluded that developing and maintaining accurate SA and effective communication are crucial for a successful prehospital MCI response, emphasizing the need for specific training and mental preparation for EMS personnel and medical incident commanders [59]. In contrast, considering specific high-threat scenarios, in 2021, Argintaru et al. focused on hospital preparedness for active shooter (Code Silver) events using in situ simulations. The identification of latent safety threats, including inadequate SA outside clinical areas, underscored the critical role of SA in these dynamic and high-risk situations. The study demonstrated the value of simulation in revealing vulnerabilities and providing a framework to enhance hospital preparedness for such incidents, emphasizing the need for comprehensive SA across all operational areas [60].

Finally, Walsh et al.'s 2015 study on healthcare coalitions (HCCs) revealed a need for better training, communication, and resource sharing to enhance disaster preparedness among health professionals, with a key focus on improving SA across member organizations [61]. While HCCs show potential, sustained effectiveness requires widespread support and shared resources for learning. This aligns with Westman et al.'s 2024 review, which emphasized the crucial role of non-technical skills, especially SA, for medical professionals in disaster response, advocating for more research into their training and implementation to improve overall disaster and emergency management [62].

### 3.2. Fostering a disaster mindset through training and education

Longo's study in a seismically active region revealed significant gaps in disaster preparedness and self-protective knowledge among nursing students, with most feeling unprepared for earthquakes and lacking basic measures despite some awareness of correct actions. This lack of personal preparedness in future healthcare professionals highlights the urgent need for targeted educational interventions to ensure they can effectively develop proper DMS, being ready to care for others and protect themselves during and after disasters [63]. Such preparedness may be achieved by simulation training as described by Carenzo et al. [51]. They describe a disaster drill designed to train anesthesiology residents in managing MCIs, addressing the often-limited disaster medicine training in this specialty. The comprehensive drill combined theory with a full-scale simulation, yielding positive results like good triage accuracy and valuable feedback on the challenges of crisis management roles. This emphasizes the effectiveness of practical training in preparing medical professionals for complex disaster response and highlights the need for experiential learning to bridge the gap between routine practice and MCI demands, creating the needed DMS [51].

The need for integrating disaster preparedness extends beyond physicians and nurses to other vital healthcare professionals, as emphasized by Glotzer et al., who advocate for incorporating disaster preparedness into dental education [64]. Dentists, with their medical knowledge and skills, can significantly contribute to public health during disasters, provided they receive specific training beyond their routine clinical practice. The paper proposes integrating preparedness elements into the existing dental curriculum and adding a dedicated capstone course on all-hazards preparedness. This aligns with the recent introduction of the Flexible Surge Capacity concept [65]. By highlighting the successful example at the New York University College of Dentistry and suggesting collaborations with various agencies, both papers underscore the importance of a comprehensive and collaborative approach to ensure all healthcare professionals are ready to contribute during crises.

The benefits of simulation training for disaster preparedness are not limited to healthcare professionals; Khorram-Manesh et al. evaluated a simulation training program aimed at increasing young students' awareness and preparedness for disasters. The EMPTY program, utilizing interactive methods to simulate a school fire and a school shooting, demonstrated significant improvements in students' personal and situational awareness, engagement, and confidence in managing emergencies [66]. The study's findings highlight the effectiveness of early and engaging educational interventions in fostering preparedness and mental readiness among youth, suggesting that cultivating a culture of preparedness should begin early in the educational system.

The adaptation of successful strategies from one field to another can also enhance disaster preparedness. Remick et al. explored how military surgical lessons can improve civilian surgeons' readiness for mass casualty events (MCEs). Military surgeons' effective practices, like Military Damage Control Surgery (integrating clinical principles with combat situational awareness in resource-limited settings), can be adapted to civilian contexts. A curricular module incorporating interdisciplinary learning at a civilian medical school showed significant knowledge gains and positive student feedback. This suggests that leveraging expertise and protocols from fields experienced in high-stakes, resource-constrained scenarios can significantly enhance disaster response capabilities in civilian healthcare [47].

## 4. Discussion

This study underscores the critical and interconnected roles of SA and DMS across the emergency response spectrum. Fundamentally, SA is the cognitive process of perceiving the environment, interpreting relevant information, and projecting future states, involving continuous data acquisition and analysis to inform decision-making regarding potential risks and hazards. Robust SA in emergency response is paramount for effective coordination, risk mitigation, and preserving life through prompt and appropriate actions grounded in a comprehensive understanding of the evolving situation [17],[40]. Key conceptualizations of SA include Endsley's three-level model (perception, comprehension, and projection) [16],[17] and Boyd's OODA loop (observation, orientation, decision, and action) [67]. Furthermore, technology augments SA through real-time monitoring tools (e.g., Geographic Information Systems (GIS)), advanced data analytics, and rapid communication platforms such as Critical Event Management (CEM) strategies, empowering emergency managers to maintain informed perspectives and enhance decision-making [5],[46],[68]. Thus, SA in emergency management is a multifaceted competency involving continuous environmental perception, thorough comprehension, and the capacity for future projection, all indispensable for effective crisis responses.

DMS involves a proactive and psychologically resilient approach to crises, extending beyond physical preparations to encompass readiness, survival, and thriving during and after disasters by mitigating fear and uncertainty. This mindset includes foresight and the ability to promptly mitigate, prepare for, respond to, and recover from disasters. Key attributes of a DMS include: 1) Resilience – the ability to recover from challenges and grow stronger through adversity; 2) Emotional Regulation – the capacity to manage and express feelings appropriately to maintain calm and focus under stress; 3) Problem-Solving Skills – the ability to identify solutions and take effective action during crises; and 4) Personal Agency – the belief in one's ability to influence outcomes, empowering proactive preparedness and response efforts [18],[64].

However, several psychological biases can impede the development and maintaining proper DMS. The optimism bias, characterized by the belief in a lower likelihood of experiencing adverse events, can lead to complacency. The normalcy bias involves underestimating disaster likelihood and impact based on past experiences. Finally, denial, a defense mechanism against anxiety, can prevent the acknowledgment of risks and necessary precautions [69]. Overcoming these barriers requires education and awareness initiatives that foster a culture of preparedness, address these biases, and promote proactive behavior. Training should cultivate a proactive and resilient mental state, empowering individuals to take ownership of their safety and contribute to community resilience by promoting risk awareness, breaking down preparedness into manageable steps, and framing it positively and empowering. In summary, a DMS is characterized by foresight, preparedness, resilience, and effective coping with uncertainty and adversity. Cultivating this mindset involves addressing psychological barriers and leveraging training and education to empower individuals and communities for effective disaster response.

**Figure 2.**
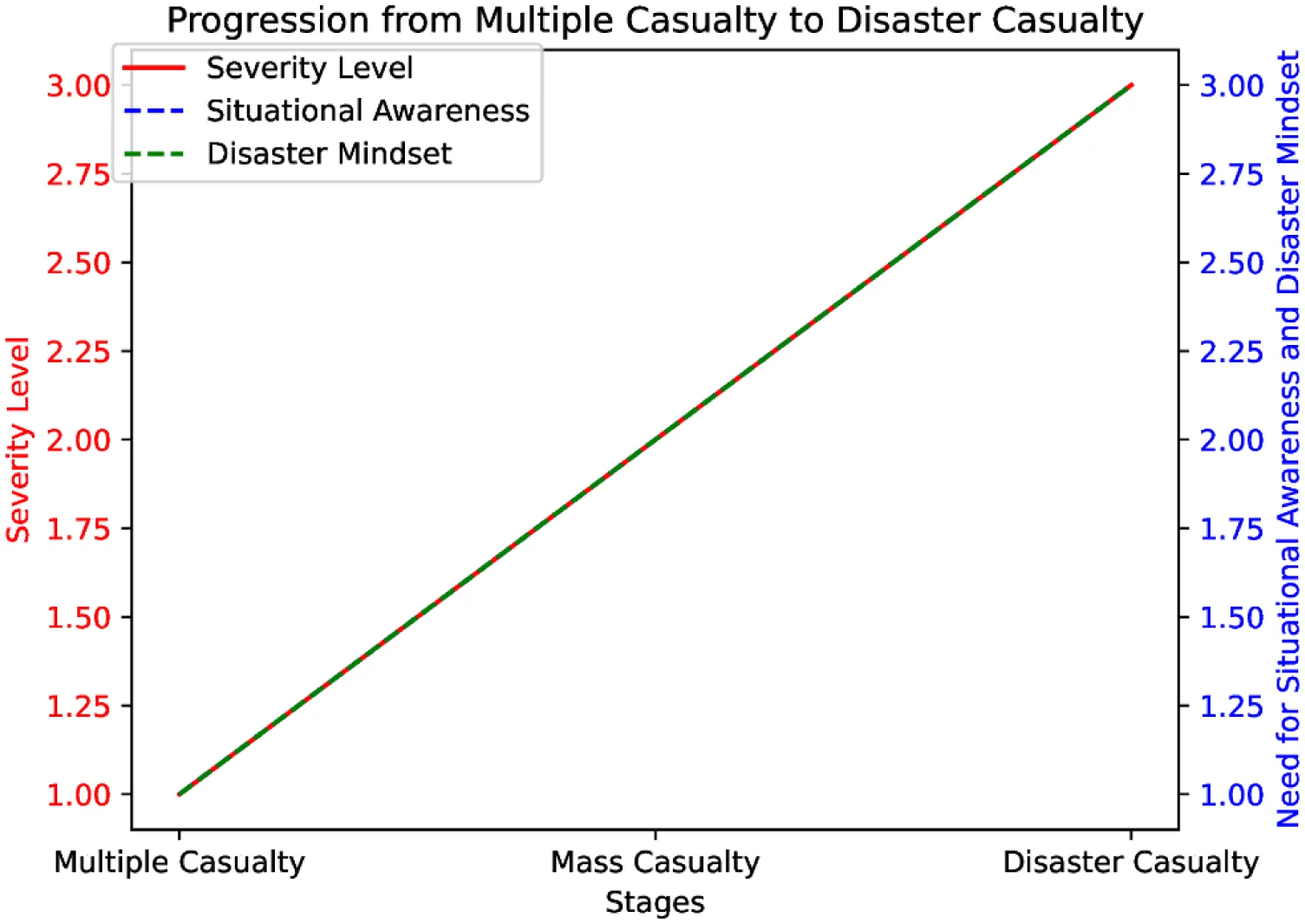
This graph helps visualize how the severity of an event (left) escalates and how the need for situational awareness and a disaster mindset becomes more critical (right) as time progresses and the situation transitions from multiple casualties to disaster casualties (baseline). Note: Event Severity: 1 = Multiple Casualty, 2 = Mass Casualty, 3 = Disaster Casualty. SA/Mindset Need: 1 = Low, 3 = High.

A nuanced understanding of these distinctions and the transition of an event into the next severe stage is necessary to design educational programs that enable emergency responders to tailor their operational approach to the specific demands of each incident type, ensuring contextually proper actions (Table 1 and Figure 2). However, current disaster education often relies on theoretical presentations and struggles to create simulation exercises that accurately assess participants' real-time reactions, SA, and DMS under realistic conditions [34]. This limitation stems from a lack of standardized disaster medicine curricula [9], educators lacking specialized training [70], a scarcity of practical, hands-on training such as realistic simulations [71],[72], insufficient emphasis on interdisciplinary collaboration [29],[30], limited integration of advanced technologies like virtual and augmented reality [73]–[75], a lack of rigorous evaluation methods [75],[76], a frequently limited global perspective [29],[30],[77], and resource constraints [78].

While disaster exercises typically focus on core pillars like planning, response, safety, triage, clinical competence, psychological first aid, interdisciplinary collaboration, and quality improvement [79]–[81], the dynamic transition from MUCIs to MCIs and DCs underscores the necessity of explicitly incorporating SA and DMS as critical additional competencies. Given the diverse actors involved in disaster and emergency management, there is a need for interdisciplinary and transdisciplinary approaches to training, education, and research [28]. For instance, disaster triage differs from that of multi- and mass casualty events. In other words, it is the same subject but uses a different approach [82]. The absence of adequate training in these cognitive and strategic domains can significantly compromise the effectiveness of established core pillars.

Beyond inherent gaps in current disaster education, simulation exercises, while valuable for training and preparedness, exhibit several key shortcomings. These include a lack of standardized procedures [81],[83], a lack of realism in capturing disaster complexity and unpredictability [84],[85], a limited range of diverse scenarios often neglecting rare but high-impact events [85], inadequate integration of interagency collaboration [86],[87], the resource intensiveness of high-quality simulations coupled with limited technology integration [88],[89], and a lack of proper oversight and standardized evaluation, which results in a disconnect between learning and assessment, potentially leading to unprepared responders in real-world disaster scenarios [90].

These statements are aligned with our analysis of strengths and converging themes in the current disaster education. Across emergency scenarios, including MCIs, mass gatherings, hospital readiness, rural healthcare, and public health crises, the literature consistently highlights the critical roles of well-developed SA and proactive DMS as fundamental for effective response. Initial assessments reveal preparedness deficits, underscoring the need for targeted educational interventions [51],[64],[66]. Research emphasizes that SA is multifaceted, requiring role-specific information, shared understanding within teams, continuous updates, and future projections. Technological solutions enhance the speed, accuracy, and accessibility of critical information for improved disaster response [44],[46],[54],[56],[57],[64].

Cultivating robust SA and a proactive DMS is strongly linked to dedicated training protocols and immersive simulations, which improve individual and team performance, identify vulnerabilities, and enhance personnel confidence [60]–[64]. The reviewed studies also delineate context-specific challenges in rural healthcare and the prehospital phase of MCIs, emphasizing the need for nuanced intervention strategies. Cohesive teamwork and interagency collaboration, particularly within HCCs and crisis management teams, are crucial, with shared SA and effective communication being key enablers [51],[54],[58],[59],[66]. Simulation drills and interactive learning enhance preparedness, knowledge retention, and responder self-efficacy, highlighting the importance of experiential learning tailored to specific professional roles. Furthermore, the adaptation of military surgical principles to civilian medical education and early preparedness education for schoolchildren demonstrate pathways for enhancing societal resilience [47],[66].

While the reviewed studies robustly advocate education and targeted training to enhance disaster preparedness across various professional groups, several limitations and areas for future research are evident. Longo's static assessment calls for longitudinal studies to evaluate the lasting impact of educational interventions [63]. Furthermore, there is a need to move beyond measuring knowledge and perceived readiness to directly assess actual behavioral adaptations and performance during real-world disaster events, as the ecological validity of simulation proxies has inherent limitations. The long-term sustainability and integration of preparedness initiatives into professional development frameworks also require more attention, along with a comprehensive consideration of the resource implications associated with intensive training methods [64].

Although improved professional preparedness is posited to enhance patient care, direct empirical evidence establishing a causal link to measurable patient outcomes during actual disasters is largely lacking. Further research should explicitly explore the psychological impact of disasters on responders and the efficacy of preparedness training in mitigating these effects, building upon observations of participant overwhelm during simulations [51].

Methodologically, while researchers employ varied approaches, future research could profitably entail SA and DMS across a broader range of responders, delve deeper into human factors influencing SA under stress, and address the pragmatic challenges of widespread technological integration. The development of standardized metrics for measuring SA and DMS in real-time remains a significant challenge, and greater emphasis on implementation science is needed to translate research findings into tangible operational and policy changes. Finally, more scholarly attention should be given to evaluating the long-term impact and ensuring the sustainability of improved SA and DMS within emergency response organizations [51].

Addressing these identified gaps, potentially through the innovative application of artificial intelligence (AI) and other advanced technologies, is crucial for advancing disaster medicine education [68],[85],[88],[89]. The outcomes of the included studies collectively underscore the vital role of targeted and experiential education in enhancing disaster preparedness across various groups. Moreover, while they demonstrate the effectiveness of specific interventions like simulation and cross-disciplinary learning, they also highlight the need for more research on long-term impact, real-world application, sustainability, and the broader integration of disaster preparedness into education and professional development.

Ultimately, the goal is to translate increased knowledge and confidence into more effective and resilient disaster response capabilities that improve outcomes for responders and affected populations. Special attention should be given to the transition between levels of incident severity from multiple casualties to disaster casualty incidents (Table 5). Such a teaching approach enables achieving the final and often missing disaster casualty stage, which better clarifies the need for SA and DMS in disaster education. Table 5 suggests diverse teaching approaches in different scenarios.

### 4.1. Recommendations

Actionable recommendations, based on this review, include individuals actively engaging in personal preparedness (plans, kits, training) and developing mental readiness. Teams should regularly conduct simulations focused on communication, coordination, and shared SA, emphasizing clear roles. Organizations should invest in user-friendly SA technologies, develop comprehensive response plans incorporating lessons learned, and foster a culture of preparedness through regular training and awareness campaigns promoting skills and DMS.

Policymakers should support research, promote disaster preparedness education in professional curricula, facilitate interagency collaboration, and consider incentives for the widespread adoption of preparedness measures. A holistic approach combining technology, targeted training, and a focus on both SA and DMS is essential for enhancing overall preparedness and mitigating the impacts of future disasters.

**Table 5.** Teaching approaches in multiple-, mass-, and disaster casualty incidents suggested based on this review.

**Aspect**	**Multiple casualty**	**Mass casualty**	**Disaster casualty**
**Focus on Scale and Complexity**	Handling a moderate number of casualties with local resources.	Managing a large number of casualties that overwhelm local resources.	Addressing a broad range of scenarios, including natural and human-made disasters.
**Triage Systems and Protocols**	Standard systems like START.	Advanced systems like SALT and MUCC.	Combination of triage systems, adapting to specific challenges.
**Resource Management**	Efficient use of local resources.	Managing scarce resources, requiring external support.	Resource management for immediate response and long-term recovery.
**Environmental Considerations**	Controlled environments like traffic accidents or smaller fires.	Complex, chaotic environments with additional hazards.	Wide range of environmental factors, including extreme weather and infrastructure damage.
**Coordination and Communication**	Local coordination among emergency services, hospitals, and stakeholders.	Extensive coordination among multiple agencies, including federal and state emergency management.	Coordination for both immediate response and long-term recovery, involving various agencies.
**Preparedness and Drills**	Regular drills and exercises for moderate casualty incidents.	Large-scale drills and simulations for mass casualty incidents.	Variety of drills and simulations for diverse disaster response and recovery challenges.

Note: START: Simple Triage and Rapid Treatment; SALT: Sort, Assess, Life-Saving Interventions, Treatment/Transport; MUCC: Model Uniform Core Criteria for Mass Casualty Triage.

### 4.2. Future research directions

- Develop and validate reliable tools for measuring SA and DMS in simulations and real disasters.
- Investigate cognitive and emotional influences on SA and decision-making under disaster stress.
- Address practical challenges of adopting new technologies in emergency response (interoperability, training, cost).
- Apply frameworks to effectively translate research into practice and policy changes.
- Examine the long-term impact and sustainability of SA and DMS improvement interventions.
- Explore SA and DMS across professional groups, organizational levels, and nations to understand interdisciplinary and international collaboration.
- Address ethical considerations of technology in SA (data privacy, security, and biases).
- Investigate factors influencing disaster preparedness behaviors beyond knowledge and attitudes.
- Develop metrics to assess the impact of preparedness education on patient outcomes in real disasters and an education infographic tool or checklist, including SA and DMS.
- Evaluate the cost-effectiveness of different preparedness education interventions.
- Explore best practices for sustainably integrating disaster preparedness education into training programs.
- Research how preparedness education can enhance responder psychological resilience.
- Investigate the effectiveness of broader community disaster preparedness initiatives.

### 4.3. Limitations of the study

The main limitation is the scoping review method. Unlike systematic reviews, a scoping review does not assess the quality or bias of included studies, potentially affecting the findings. In addition, our broad scope, aiming for an overview than in-depth analysis or specific answers, may lead to diverse studies complicating synthesis. It is also acknowledged that some programs may incorporate elements of SA and/or DMS without naming as such.

While systematic, researcher judgment in selection and extraction could introduce bias, mitigated by three independent collaborators. Scoping reviews are descriptive and exploratory, not for quantitative/qualitative synthesis or intervention effectiveness. Though potentially less resource-intensive than systematic reviews, they require significant effort and are not ideal for very specific questions. However, this broad approach suited the study's goal of establishing a foundation for future, focused research.

## 5. Conclusions and implications

For effective emergency response, understanding what is happening (Situational Awareness or SA) and having the right mental approach (Disaster Mindset or DMS) are crucial and work together. SA is being improved by technology. DMS needs training to overcome natural biases. By focusing on realistic training, using technology wisely, and educating people effectively, we can build SA and DMS, making individuals and organizations better prepared to handle disasters and reduce their impact.

Key implications for practice emphasize investing in user-friendly technological solutions to enhance real-time SA through tools like RFID, UAVs, and SMS. Moreover, realistic, simulation-based training across professions is vital for improving SA and fostering proactive DMS, offering safe environments to apply knowledge and build confidence. Such training development needs to be adaptable to low-income/low-resource settings.

Furthermore, addressing persistent communication challenges through improved protocols and technologies is essential for effective coordination, especially in multidisciplinary teams during initial MCI response. Targeted educational programs are needed to address preparedness gaps in various populations, emphasizing response skills and personal preparedness. Finally, explicitly integrating the concept of DMS and encompassing psychological preparedness and resilience into all levels of disaster training is crucial for equipping individuals with the mental fortitude to cope with crises.

## Data availability statement

The original contributions presented in the study are included in the article/supplementary material; further inquiries can be directed to the corresponding authors.

## Use of AI tools declaration

The authors declare they have not used Artificial Intelligence (AI) tools in the creation of this article.


